# Stakeholder perspectives on barriers and enablers to recruiting anxious children undergoing day surgery under general anaesthetic: a qualitative internal pilot study of the MAGIC randomised controlled trial

**DOI:** 10.1186/s13063-021-05425-z

**Published:** 2021-07-16

**Authors:** Jennifer Kettle, Chris Deery, Robert Bolt, Diana Papaioannou, Zoe Marshman

**Affiliations:** 1 School of Clinical Dentistry, University of Sheffield, 19 Claremont Crescent, Sheffield, S10 2TA UK; 2 School of Health and Related Research (ScHARR), University of Sheffield, Sheffield, UK

**Keywords:** RCTs, Qualitative, Paediatric Anaesthesia, Paediatric Anxiety

## Abstract

**Background:**

The ‘Melatonin for Anxiety prior to General anaesthesia In Children’ (MAGIC) trial was designed to compare midazolam and melatonin as pre-medications for anxious children (aged five to fourteen), undergoing day-case surgical procedures under general anaesthesia. Low recruitment is a challenge for many trials, particularly paediatric trials and those in ‘emergency’ settings. A qualitative study as part of MAGIC aimed to gather stakeholder perspectives on barriers and enablers to recruitment.

**Methods:**

Sixteen stakeholders from six sites participated in semi-structured interviews about their experiences of setting up the MAGIC trial and recruiting patients as part of the internal pilot. Data was analysed using framework analysis.

**Results:**

Participants identified barriers and enablers to recruitment. Barriers and enablers related to the study, participants, the population of anxious children, practitioners, collaboration with other health professionals, ethics, specific settings and the context of surgical day units and the wider health system. Attempting to recruit anxious children from a surgical day unit is particularly challenging for several reasons. Issues include the practicalities of dealing with a child experiencing anxiety for parents/guardians; professional unwillingness to make things more difficult for families and clinicians and nurses valuing predictability within a busy and time-sensitive setting.

**Conclusions:**

Multi-site RCTs face recruitment barriers relating to study-wide and site-specific factors. There are multiple barriers to recruiting anxious children due to undergo day-case surgery. Barriers across domains can interrelate and reinforce each other, reflecting challenges relating to populations and settings. For example, in the case of anxious children, parents and other health professionals are concerned about exacerbating children’s anxiety prior to surgery. They may look for ways to keep things predictable and avoid the uncertainty of an RCT. Pre-trial engagement work could help address concerns among collaborating health professionals.

Using rapid ethnography during set-up or an internal pilot to focus on how the protocol will be or has been operationalised in practice may help identify issues. Allowing time to reflect on the findings of internal pilots and implement necessary changes could facilitate higher recruitment during the main phase of a trial.

**Trial registration:**

NIHR Trial Registration Number: ISRCTN18296119. Registered on October 01, 2019.

**Supplementary Information:**

The online version contains supplementary material available at 10.1186/s13063-021-05425-z.

## Background

High-quality clinical research is necessary to improve patient care. Well-designed randomised controlled trials (RCTs) are considered as a gold standard in achieving this high quality through addressing risks of bias and confounders. However, trial recruitment often fails to meet targets, and extensions are often required [1, 2]. Poor recruitment can lead to the discontinuation of trials, wasting resources and delaying publication of the evidence required to improve patient care [3]. Barriers exist for patients (e.g. additional demands of the trial, treatment preferences and worry caused by uncertainty) and health professionals (e.g. insufficient resources, concerns about patients and loss of clinical autonomy) [4]. Those designing trials may also overestimate the numbers of eligible patients or not consider the logistical challenges of particular research contexts [5]. Qualitative research has been used to explore why recruitment is often difficult, highlighting both ‘clear obstacles’, such as time constraints, and ‘hidden challenges’, such as role conflict [6, 7].

Using qualitative research in the pilot phase of an RCT can provide insights into recruitment challenges and improve recruitment in the main trial [8–10]. As well as understanding why patients do and do not agree to participate in RCTs, recruitment needs to be considered from the perspective of stakeholders, such as clinicians, research nurses and those involved in designing the trial. Qualitative research on stakeholders’ experiences highlights the challenges involved in operationalising the trial protocol into usual practice, fully understanding the intervention, and issues relating to professional roles [6, 11–15]. Barriers and enablers to recruitment relate to various factors, including those among patients, practitioners and within particular settings, as has been identified by French and Stavropoulou and Team and colleagues [12, 15]. The need to engage with clinical colleagues not directly related to the trial is also a recognised issue [12, 16, 17].

RCTs take place in a range of primary and secondary care settings and may involve a variety of patient populations. Each setting and population poses its own challenges, and a lack of experience specific to that population can be a barrier to recruitment [18]. Parental and health professional concerns about protecting children, due to their vulnerability, can make paediatric trials a particular challenge [19, 20]. Exploring barriers and enablers to recruitment in different contexts contributes to a better understanding of the ways in which specific and general challenges arise for clinicians and other health professionals in research-focused roles. This paper highlights particular challenges around recruiting anxious children about to undergo a general anaesthetic and conducting a study in a surgical day unit. In this paper, we focus on the experiences of health professionals in order to contribute specifically to the literature on stakeholder perspectives on recruitment. The views of parents and children will be discussed in the trial report.

### The MAGIC trial

Anxiety at the point of anaesthetic induction is a significant issue in paediatric patients, which can lead to non-compliance and is also associated with greater post-operative pain, agitation and sleep disturbance following surgery [21, 22]. Midazolam, the current standard pre-medication given to an anxious child prior to surgery, is effective, although has various adverse effects in the short-, medium- and long-term [23–26]. Melatonin is a functionally diverse hormone with anxiolytic properties (confirmed in the adult population) and many potential benefits [27]. Trials assessing the effects of melatonin in children have produced varied results [28–32], and there is a need for a definitive pragmatic trial with anxious children.

The Melatonin for Anxiety prior to General anaesthesia In Children (MAGIC) RCT is a UK-based parallel group, single blind, individual participant-randomised, stratified, multi-centre trial. The trial was designed with the primary objective of comparing the efficacy of melatonin and midazolam as pre-medications for anxious children prior to a general anaesthetic, using the modified Yale Preoperative Anxiety Scale (mYPAS) at three measurement points (start of transfer to theatre, entry to theatre and administration of anaesthesia) [33]. Secondary outcomes include comparisons of safety outcomes, efficacy outcomes, acceptability and cost-effectiveness.

In the pilot phase, participants were children aged five to fourteen requiring day-case elective ear, nose and throat (ENT), opthalmological or dental surgery under general anaesthesia. Inclusion criteria were pragmatically assessed as ‘requiring pre-medication for high/expected high levels of preoperative anxiety and being ASA grade I or II’. Patients with a current prescription of melatonin, midazolam or other medication known to interact with either trial drug, patients with obstructive sleep apnoea and patients with a severe learning disability rendering the child unable to communicate were excluded. Parental consent was required, and children were required to be willing to assent.

As part of the recruitment process, patients were screened at both pre-assessment clinics and on the day of surgery, subject to the circumstances surrounding their anxiety. If patients were judged as requiring a pre-medication on the day of surgery, they were invited to be recruited into the MAGIC trial and following consent, randomised. Either 0.5mg/kg of midazolam or melatonin was administered orally at 30 min (±10 min) prior to anaesthesia. Patients were observed by a blinded research nurse through the preoperative period until anaesthetic induction and then monitored postoperatively until discharge. Patients were then followed up 14 days after discharge by a research nurse or anaesthetic/surgical trainee.

MAGIC researchers aimed to recruit 624 patients to provide 90% statistical power (allowing for 5% attrition). At the end of the 6-month internal pilot, fewer than the target of 78 participants had been recruited. A qualitative project conducted alongside the pilot phrase was used to identify barriers and enablers to recruitment. In this article, we present the main barriers and enablers to MAGIC recruitment and contribute to the development of a framework of factors that influence recruitment across all RCTs.

## Methods

### Study design

During the internal pilot study, we conducted a qualitative study with stakeholders, involving those who contributed to the trial design, anaesthetists and research nurses, with the aim to identifying barriers and enablers to MAGIC trial recruitment. Qualitative research was used to collect detailed accounts of how recruitment was experienced from multiple perspectives. The internal pilot study was included within the trial design to allow changes to be made to the main phase of the trial. Interviews were also conducted with participants in MAGIC and their parents/guardians to understand recruitment from multiple perspectives, although this paper focuses on the views of stakeholders. Methodologically, the qualitative study was designed pragmatically on the basis of a 'subtle realism' (i.e. an external reality exists outside of people’s interpretations, but can only be accessed through these interpretations, not directly) [34]. Listening to the experiences of a range of stakeholders adds to the richness of our understanding of a particular situation [35]. Semi-structured interviews were used in order to focus discussions on the topic of recruitment, while allowing stakeholders to explain how recruitment was experienced from their varied perspectives. For means of description, the term ‘participant’ has been used to reference a patient recruited into the MAGIC trial; whereas the term ‘stakeholder’ has been used to reference a trial researcher who has acted as a participant in respect to this qualitative study.

### Recruitment

The principal investigators (PIs) of eight sites were approached by email, including the lead site. PIs were invited to take part in an interview and provide contact details of at least one other member of staff involved in the trial. Two stakeholders involved in the trial design at the lead site were approached directly by the researcher. Potential interviewees were informed that the aim of the qualitative study was to evaluate the MAGIC pilot from the perspective of health care professionals, focusing particularly on experiences of recruitment. One PI declined to take part in the qualitative study due to time constraints and one did not respond to multiple attempts at contact; the site subsequently stopped recruiting patients. Sites were chosen from different areas of the country to reflect the different numbers of patients screened and varying levels of success with recruitment (see Table 1).

**Table 1:** Screening and recruitment at the point qualitative study commenced (October 2019)

Site	Number screened	Number recruited	Percentage recruited	Response to contact
Site A	13	1	7.69%	Accepted
Site B	9	2	22.22%	Accepted
Site C	10	1	10%	Accepted
Site D	6	4	66.67%	Accepted
Site E	9	3	33.33%	Accepted
Site F	29	1	3.45%	Accepted
Site G	2	1	50%	PI declined to take part
Site H	23	2	8.7%	PI did not respond. Site subsequently ceased recruitment

### Ethical considerations

The qualitative study was approved by North West – Liverpool Central Research Ethics Committee (IRAS 228234). Prior to interview, stakeholders were provided with an information sheet about the qualitative study and given the opportunity to ask questions. All stakeholders provided written, informed consent, either in person or by sending a consent form to the researcher. For telephone interviews, the researcher also obtained verbal confirmed consent to record the interview. As certain stakeholders are not anonymous due to their named roles in the MAGIC trial, specific consent has also been obtained to quote from their interviews in this paper.

### Data collection

Data collection took place between November 2019 and January 2020 and involved 15 interviews with 16 stakeholders from six sites (one PI and research nurse requested to be interviewed together for convenience). Seven interviews were conducted face-to-face and eight were conducted by telephone. Face-to-face interviews took place in the stakeholders’ offices, or small private meeting rooms in the hospital. All interviews were conducted by the first author, who is female, has a PhD, is experienced in conducting qualitative research and is not a clinician. The first author was independent of the main trial team and approached stakeholders as an outsider to paediatric anaesthesia and clinical research in order to avoid making assumptions about how things worked in practice. The independence from the trial team and lack of clinical experience were disclosed at the start of the interviews. Fourteen of the stakeholders were not known to the interviewer prior to the interview; two were colleagues working in the same dental school, although previous contact was relatively limited. No repeat interviews were undertaken.

Interviews lasted on average 65 min, and ranged from 35 to 105 min. A topic guide was developed, drawing on existing research of those barriers and enablers to recruitment in RCTs (see Appendix 1). The topic guide was developed by the first author and approved by another qualitative researcher with experience in conducting interviews as part of RCTs (ZM). The topic guide was not pilot tested. Notes were made during the interviews to highlight significant points for further discussion. Interviews were audio-recorded, transcribed verbatim by an external company and checked by the first author to ensure data quality. Transcripts were not returned to stakeholders for checking. Data collection ended at a point where the intended sample (two to four stakeholders from each of five or six sites) had been recruited, in order to feedback findings to the trial team.

### Analysis

All transcripts were uploaded to NVivo 11 (QSR International) for analysis. Framework analysis was used as it provided a pragmatic approach [36] which produced results that could be easily incorporated into the trial [37]. The analysis involved the following stages: identifying initial themes, labelling the data, sorting the data by theme and synthesising the data. Transcripts were read and re-read by the first author to achieve familiarity, and an initial framework was developed, relating to aspects of stakeholders’ experiences of MAGIC. All transcripts were coded by the first author only, according to this framework. The data was then summarised in relation to the themes and sub-themes. Barriers and enablers to recruitment were identified from these data summaries. The barriers and enablers were organised, drawing on Team et al.’s existing framework of factors relating to recruitment [12, 15]. These factors have been defined further (see Table 2).

**Table 2:** Factors affecting recruitment in randomised controlled trials, adapted from Team et al. 2018

Type of factor	Working definition
Study-related factors	Design of the study, specific intervention, and attitudes and behaviours of the central trial team (in multi-site studies)
Participant-related factors	Attitudes, behaviours and capabilities of potential participants and parents (in paediatric studies)
Practitioner-related factors	Attitudes, behaviours and capabilities of clinicians and other healthcare professionals involved in recruitment
Ethics-related factors	Ethical considerations of practitioners that impact on recruitment
Collaboration-related factors	Collaboration with other healthcare professionals not directly involved in recruitment
Setting-related factors	Layout, logistical arrangements and resources of the specific sites involved
Context-related factors	Broader health speciality context in which the study was carried out
Health system-related factors	Health system in which the study took place, and the responsibilities of clinicians and research nurses within this

**Table 3:** Barriers and enablers to recruitment

Factor	Barriers	Enablers
Study-related	• Anxious patients excluded by eligibility criteria • Acceptability of intervention to patients • Delay in trial opening unhelpful • Difficulty re-engaging sites after delay • Lack of engagement with sites by CI • Information not provided in multiple languages • Lack of thinking time for parents/guardians and patients • Amount of information parents/guardians required to read • Amount of paperwork parents/guardians required to complete • Difficult to improve recruitment	• No issues with design • Straightforward trial • Clear information for parents/guardians and patients • Use of established medications • Well organised study • Funding provided for research nurses • Supportive, available study team • Study team keeping site updated
Participant- and population-related	• Fewer anxious patients than expected • Parents unwilling to attend appointments due to timing (school year) • Parents and patients declining to take part in trial • Patients preferring not to have a pre-medication • Concerns about child’s anxiety • Parents and patients unwilling to read necessary information • Limited proficiency with English • Anxious dental patients generally difficult to recruit • High levels of anxiety requiring higher levels of pre-medication • Limited anxiety in ENT and opthamology patients	• Generally positive response to MAGIC • Trial seen as helpful for patient anxiety • Parents wanting to help (NHS, other children) • Parents comfortable with either treatment option
Practitioner-related	• Lack of clinician availability • Lack of research nurse availability • Not recruiting from all specialities • Lack of experience in setting • Discomfort with investigator role • Inertia around first recruit • Lack of engagement in trial • Lack of motivation following poor recruitment • Unintentionally unhelpful actions of other stakeholders	• Trial valued by clinicians and research nurses • Personal investment of PI in MAGIC • Practitioners comfortable explaining trial • Effective communication between practitioners • Organisation and preparation • Ensuring clinician availability • Ensuring research nurse availability • Ongoing engagement with the trial • Acting to improve recruitment
Ethics-related	• Excluding patients based on clinical experience • Concern about making things more difficult for families • Concern about worsening anxiety • Concern about children who can’t give assent	• Important that assent is included • Children appreciating opportunity to give assent • Option of verbal assent is helpful
Collaboration-related	• Anaesthetists gatekeeping • Anaesthetists wanting predictable approach • Different approaches to pre-medication among anaesthetists • Lack of personal relationships with key personnel • Issues with communication at site • Difficult to engage people outside the trial • Theatre nurses gatekeeping	• Buy-in from other clinicians, health professionals • Nurses identifying anxious patients • Effective working relationships with key personnel • Engagement work to improve relationships • PI publicising trial
Setting- and context-related	• Difficult to recruit on the day • Pressure on resources • Challenge of recruiting in time-pressured environment • Limited time to decide about approaching patients • Lack of pre-assessment • Pre-assessment not working to enable recruitment • Delays getting prescriptions from pharmacy • Challenges following protocol in particular settings	• Advance notification of anxious patients • Actively identifying potential participants • Working well with existing system • Ability to be flexible with surgical lists • Effective organisation of paperwork • Effective process with pharmacy
Health system-related	• Demands of NHS workload • Limitations of research nurse system • Lack of remuneration for unseen work • Pressure on NHS resources	• Good for departments to be involved in research • Research nurses available for various studies

**Table 4:** Participant characteristics

Participant	Role	Site	Type of setting	Patients recruited at time of interview
S1	Anaesthetist, PI	B	District general hospital	2
S2	Research Nurse	B	District general hospital	2
S3	Paediatric Dentist, PI	C	Teaching hospital	1
S4	Dental Surgeon, Co-Investigator	A	Dental hospital	2
S5	Paediatric Dentist, CI	A	Children’s hospital	3
S6	Research Nurse	D	District general hospital	7
S7	Anaesthetist, PI	D	District general hospital	7
S8	Anaesthetist, sub-PI	E	Teaching hospital	4
S9	Research Nurse	E	Teaching hospital	4
S10	Anaesthetist, PI	F	Children’s hospital	2
S11	Research Nurse	E	Teaching hospital	5
S12	Anaesthetist, PI	E	Teaching hospital	5
S13	Anaesthetist (Lead Anaesthetist for Trial), PI	A	Children’s hospital	6
S14	Research Lead	C	Dental hospital	3
S15	Research Nurse	A	Children’s hospital	7
S16	Research Nurse	F	Children’s hospital	2

The identified barriers and enablers were refined within the qualitative research team (JK and ZM) (Table 3).

The findings were shared with two of the stakeholders involved in designing the trial (CD and RB) who provided feedback and contributed to the final conceptualisation of barriers and enablers.

## Results

Participants in this qualitative study comprised the Chief Investigator (CI), Co-Investigator, six PIs, an anaesthetist working as a sub-PI, six research nurses and a research lead from one of the sites involved in MAGIC (Table 4).

Stakeholders identified barriers and enablers to recruitment within MAGIC, which are outlined below, using anonymised illustrative quotations.

### Study-related factors

#### Barriers

Recruitment was limited to some extent by the eligibility criteria, which restricted patients in terms of age and clinical speciality. Some stakeholders felt that other anxious children who often received pre-medications could have been included, such as younger children:

*But three to five are the worst actually. And we’ve identified so many children like that, three to five. They're very anxious. And I'm surprised this study did not include that bit of the population.* (PI, Site D)

The protocol restricted recruitment to ENT, dentistry and opthamology specialities. However, some stakeholders felt children undergoing plastic surgery, urology and/or MRI scans also required pre-medications and could have been included. The eligibility criteria also specified day-case procedures, which excluded groups of otherwise eligible patients who were required to stay overnight, or who went to a ward that allowed for overnight stays (even thought it was expected the children would go home the same day).

The design of the study also potentially acted as a barrier, in terms of acceptability of the intervention (giving larger volumes of pre-medications as compared to standard practice owing to limitations in how the investigational medicinal product (IMP) could be manufactured in compliance with the Medicines and Healthcare products Regulatory Agency); the lack of thinking time available to potential participants if approached on the day of surgery; and the information required of parents/guardians.

*The volume is a definite big one though, 20ml is a lot to take.* (Research Nurse, Site E)

*They're not given a huge amount of time to consider the study.* (Research Nurse, Site A)

*They don't necessarily expect you to ask them if they're working and how much money they earn kind of thing […] that's questions that are probably going to add to their anxiety.* (Research Nurse, Site F)

The amount of information patients were required to read could also potentially be off-putting, and although a video for children had been provided, it was not always possible to show this if families were approached in a busy waiting area, and no side room was available. Information was also only available in English, which is not always the first language of parents/guardians.

Delays in the trial set-up for MAGIC resulted in many sites opening during the summer school holidays, when several PIs and research nurses were on annual leave, delaying the start of recruitment. The CI also found it was difficult to re-engage with sites after the delay and maintain momentum:

*We had a delay getting the drug made, so there was just a bit of a hiatus and I think we haven’t done particularly well at re-engaging people […] at just getting that oomph back in it that we had*. (CI)

The CI acknowledged this reflected on him and that he could do more to engage people (such as friendly texts, and emails to remind people about MAGIC). However, he also noted this was difficult where there was not already an established relationship with the PI.

#### Enablers

Although concerns were expressed about eligibility criteria and the situation in which patients needed to be recruited, stakeholders were positive about the design and organisation of the trial, as well as the funding for research nurse time. One potential enabler was the choice of medications; both were seen as well-established which could be reassuring to parents:

*They’re very happy to be involved particularly when you reassure them that both medications we’re giving are well-established medications that have been used in children for lots of other things.* (PI, Site B)

In general, it was also felt that the information being provided was clear, including a video designed for children.

*I think the video is a nice way of introducing it for the children and, obviously, with the standard kind of information sheets which have all been produced very nicely.* (PI, Site C)

Interviewees were all positive about the communication with the trials unit, noting that they received swift responses to questions and found the question and answer sessions useful, both for practically addressing questions and promoting a sense of a wider study community:

*They do questions and answers as well with other hospitals which is useful […] it just makes you think that you're not the only one that’s got those problems which is good.* (Research Nurse, Site D)

### Participant- and population-related factors

#### Barriers

The interviews suggest that there are fewer eligible patients than expected:

*We're still going two or three weeks and screening and there's nobody sometimes.* (Research Nurse, Site E)

Time of year potentially contributed to differences between audit figures and figures at the time sites opened. For instance, parents/guardians may avoid elective surgery early in the school year.

Some parents/guardians of eligible children declined to take part and appeared uncomfortable with the uncertainty involved, particularly given the stressful situation:

*Their parents are probably anxious and stressed enough as it is without being presented with another thing to sort of think about. But that’s just what happens when your child is about to be anaesthetised I guess.* (PI, Site E)

One site noted that there was some rejection of the MAGIC trial by older children who did not want a pre-medication.

Stakeholders also noted that anxiety can make it harder to function and take on information and that parents/guardians as well as children can be anxious when a child is due to undergo a general anaesthetic. This can be a barrier to recruitment:

*I think when people are anxious, it’s more difficult for them to function and take in information and understand the information. And I think sometimes they’re so worried about things that, actually, they can’t sit down and focus. And also, they’ve got a child to manage as well.* (PI, Site F)

Another barrier was parent/guardian ability to digest the information required to provide informed consent. For example, where English was not a parent’s first language, this could also act as a barrier to recruitment:

*We get a lot of Polish patients and that’s the language... I have seen, they're just like, they know a little bit of English but not to the extent that they can read and digest the information.* (PI, Site D)

Similarly, education levels could be perceived as a barrier:

*I guess the population we’re dealing with aren’t always that literate or educated. That’s probably fair, you know, in this part of the world. And I think it’s quite a big effort for them to come in and read everything and understand it.* (PI, Site F)

This also relates to ‘very lengthy’ (Research Nurse, Site E) information sheets, although it is acknowledged that it can be difficult to reduce the amount of information required for legal and ethical reasons.

MAGIC was recruiting patients requiring dental, ENT and opthalmology. While these specialities were chosen due to their similarities, stakeholders felt that there were differences between populations. Anxious dental patients could present a specific recruitment challenge, particularly if they had higher levels of anxiety related to dental phobia (and potentially needed a higher dose of pre-medication than allowed within MAGIC) and had less understanding of what to expect:

*They're coming to hospital because they're going to get a dental procedure which they're already scared of. It's just a really fine balance to strike. The children need to have severe anxiety to be eligible for the study but there's also an anxiety level that's probably too severe for our study.* (Research Nurse, Site F)

To some degree more of the kids are coming from lower socioeconomic groups because of dental caries, maybe have less supportive parenting backgrounds or parental prep, you know, so they sometimes come less informed about what’s going to happen to them. (PI, Site E)

In contrast, ENT and opthalmology patients were often found to be less anxious, with lower numbers of pre-medications required:

*An ENT pre-med is quite rare, they just don't seem to happen as much.* (Researcher, Site C)

#### Enablers

Some stakeholders reported a largely positive response from parents/guardians, which is a potential enabler:

*They're very welcoming […] Because I think every parent likes their child to be less anxious coming to the theatre.* (PI, Site D)

This seemed to relate to a lack of concern about the two pre-medications, and the potential that participating would help with their child’s anxiety. Parents/guardians also saw participating in the trial as a chance to give back to the NHS.

### Practitioner-related factors

#### Barriers

The main issue for recruiting clinicians was availability. Clinicians were not able to recruit at certain times, due to other commitments:

*There were patients that we’re probably missing because I was on holiday or I just wasn’t there, and therefore because the people who were anaesthetising when we were away weren’t listed as the investigators then they weren’t able to do recruitment.* (PI, Site E)

Similarly, research nurses were not always available to recruit, as they generally worked on multiple studies:

*We just need to be good here that if I have other patients booked in that the backup nurse comes over that day and unfortunately, if both of us are busy there's no one to go over and screen and recruit patients.* (Research Nurse, Site F)

Some PIs had decided to focus recruitment on particular specialities (e.g. not recruiting opthamology patients) in order to make the best use of resources, but eligible patients may have been missed as a result.

Several research staff reported a lack of experience attempting to recruit patients on surgical day units. Although research nurses are professionals who can draw on wider experience of recruitment, a lack of experience in this particular setting may be a barrier, at least initially. There was some acknowledged nervousness around the emotional challenges and unfamiliarity:

*It's quite a distressing environment seeing children upset […] so I think emotionally you have to try and detach yourself a little bit. And we're not necessarily used to doing that. So yeah, we've all been, sort of, very nervous about it.* (Researcher, Site C)

Clinicians may display initial ‘inertia’, being reluctant to recruit when the protocol is still relatively unfamiliar:

*I know myself, the first time you have to take someone through a consent process […] it seems like a bit of hassle and you’re busy so you don’t...you think, “I’ll do it for the next one, I won’t do it for this one.” […] I think there’s inertia as well happening to people.* (CI)

Over time, lower than expected recruitment, despite significant effort, could be demotivating:

*It is quite disheartening when you're not getting anywhere with the patients or you just get constant noes and you're really at a loss as to how you can improve.* (Research Nurse, Site F)

Stakeholders also identified how the actions of other people involved in the trial could be a barrier; for example, a PI who was unwilling to involve other anaesthetists in recruiting participants, resulting in patients being missed, or an anaesthetist who presented the side-effects of midazolam to a parent in an off-putting way.

#### Enablers

MAGIC was widely seen as a valuable study, and stakeholders were keen to recruit. Individual PIs being personally invested in the results of a trial may be an enabler to recruitment:

*I wanted to be involved because I think the information, if we can get a full trial and it does come up positive, it would make my job easier.* (PI, Site B)

For example, this PI read up on melatonin to ensure she was fully informed, actively took the lead in terms of setting up and running the trial and planned on paying recognition to those staff members who recruited the most into the trial.

As noted above, MAGIC was seen as straightforward, and both clinicians and research nurses were comfortable explaining the trial and answering questions. Clinicians highlighted how they demonstrated equipoise, but also indicated both pre-medications are established drugs, while research nurses showed how they could discuss the trial in an accessible way:

*[I’ll] use child-friendly terms like if your tummy feels like a washing machine or you have butterflies in your tummy […] And sometimes these medicines what they'll do is they'll help settle your tummy so it'll feel nice and calm and you won't have that feeling but, you know, it can also make you a little bit sleepy as well so you're nice and relaxed. And the kids seem to be quite happy with that.* (Research Nurse, Site F)

Stakeholders felt that good communication helped to facilitate recruitment:

*I think just keeping it as a small team […] we can communicate amongst us just so that we’re all aware of the patients or the potential patients that could be recruited.* (Anaesthetist, Site E)

PIs made sure they or another clinician or trainee were available to confirm eligibility. Recruitment was also felt to be enabled by the availability of key people when anxious patients arrive.

When combined with the advance notification, research nurses were able to use organisational skills to manage their time in order to ensure availability, or arrange cover:

*For myself as a research nurse involved in lots of different studies, as soon as I get that email, I allocate that time in my diary, and it’s there and I know that that’s where I need to be.* (Research Nurse, Site B)

Although demotivation is seen as a barrier, recruitment could be facilitated by ongoing engagement and a willingness to act to increase recruitment. Participants reported meeting to discuss ways to improve recruitment, adding more clinicians to the delegation log and setting up better communication systems:

*We're working on the logistics […] we have a WhatsApp group so we know who's available every week in advance and every day of the week who will available for recruitment.* (PI, Site A)

### Ethics-related factors

#### Barriers

Participants were balancing their responsibilities as clinicians/nurses and researchers. In some cases, eligible patients were not recruited due to concerns about whether the specific medications would be effective for the individual, for example in the case of children over 60kg , where outside of the trial a 20mg ceiling dose of midazolam may not have applied:

*It’s just not ethical to include them if you know what you’re going to give is not going to be effective if they got a midazolam portion.* (PI, Site B)

From a trial design perspective, 0.5mg/kg to a maximum dose of 20mg related to solubility and limitations on the volume of liquid that could be given as a premedication (20mls as a maximum), and it was accepted that this would exclude heavier children.

Research nurses were also concerned to avoid adding to patient distress and making things more difficult for families:

*As soon as they came in, the father actually did not seem well and the child is screaming and just was not appropriate to go near them at all, because they were overly anxious.* (Research Nurse, Site E)

In this case, the child had autism, and there were other concerns expressed about approaching children with learning and developmental disabilities, and increasing the burden on families.

There was also a concern that trying to recruit patients could add to anxiety, both by drawing attention to the fact they would be having a pre-medication and taking parents’ attention away from the child:

*If it's another 15, 20 minutes to go through paperwork with the parents, that's 15, 20 minutes of the child being more worked up.* (Research Nurse, Site F)

The requirement for assent can be seen as a barrier to recruitment, if children do not fully understand what is involved. This was an issue for children with severe learning disabilities, who could be anxious but judged unable to communicate with the researchers.

#### Enablers

Although obtaining assent can be difficult, stakeholders saw it as important and viewed the option for verbal assent as helpful. There were concerns about the possibility of dropping the requirement for assent, particularly where this was not found to be a barrier:

*I think that you're asking the child to take the medications, and I think GCP (Good Clinical Practice) and it's the right of the child that they should be able to, if they can't do it written then they should at least be able to say "I'm happy to take part in this trial and I understand."* (Research Nurse, Site E)

### Collaboration-related factors

#### Barriers

Collaboration was an issue in this study as participants often relied on anaesthetists outside of the trial to give permission for their patients to be recruited. Participants reported that some anaesthetists were unwilling to allow this, suggesting it would be in the best interests of the patients to continue with a more familiar practice:

*I think their feeling of that with their experience, they want to maintain their own practice because they feel that that is going to be more successful for the patient. So honestly, they’re meeting the patients and speaking to the parents and, you know, I think they feel responsibility to make sure that they succeed at getting it done.* (PI, Site F)

This reflected preferences for other pre-medications and non-pharmacological approaches:

*Other anaesthetists used a combination of other treatments, so they might use midazolam and clonidine and they don't want to—they don't want any of their patients to have anything apart from that.* (Research Nurse, Site E)

*We have one anaesthetist who really doesn’t do very many pre-meds at all […] his phrase is – “I’m the pre-med” and actually, he’s really good with children and he usually manages to do them, just using his own kind of non-pharmacological techniques I suppose he would say.* (PI, Site C)

It was also noted that some anaesthetists may perceive melatonin as a ‘less potent drug’ (PI, Site E) and that as an alternative to midazolam, melatonin could be in competition with dexmedetomidine, a new drug which is ‘becoming popular’ (PI, Site A), as a result some anaesthetists may be unwilling for their patients to be recruited to MAGIC.

In larger hospitals, the requirement to work with a number of staff in the setting of the surgical day unit could be challenging and ‘chaotic’ (Research nurse, Site A). In relation to this, it was suggested that at a larger site such as Site A, not all anaesthetists were aware of the trial, which could affect recruitment. PIs sometimes found it difficult to engage clinicians due to the work involved (e.g. completing General Clinical Practice (GCP) training):

*To get, somebody to get their GCP up to date, and get a CV together, a research CV, and various other bits and bobs. It doesn’t sound like a lot of work to do to actually register. But I think for some people, it’s just, you know, it’s just not top of their priority lists.* (PI, Site F)

Researchers also collaborated with theatre nurses, who could request that children were not approached:

*Sometimes the theatre nurses, the standard nurse who’s not involved in the trial particularly, who had seen the patient and kind of checked them in, would say, “Oh please don’t approach that patient, our lives have already been really difficult today with them anyway, we don't want to make things any more difficult.”* (PI, Site C)

As with the anaesthetists, it was perceived that nurses could act as gatekeepers, effectively excluding eligible patients from being approached about the trial, in order to act in their best interests.

#### Enablers

Some stakeholders felt there was a strong sense of team buy-in from other health professionals:

*The nursing staff on the ward even though they’re not doing the monitoring, they know why I’m there, they’re giving the IMP, they know the value of the study so they’re on board.* (Research Nurse, Site B)

Whereas a lack of relationship could be a barrier, some participants drew on existing relationships with nurses working on surgical wards and felt this enabled recruitment:

*I think as well it works because I have worked down on surgical unit so I know all the staff down there […] now as I'm going down, they’ll say “we’ve got a patient for you”, a potential patient, “we think this one’s anxious,” so we’ve got a good team.* (Research Nurse, Site D)

Recruitment could also be facilitated by engagement work in advance (allowing staff to get to know researchers and ask them questions) and building rapport during the course of the trial:

*As long as you allow them to ask the questions and you answer them as best you can and you realise that it's a big ask because they're a busy department, often under-resourced, et cetera […] You get on to a better level of communication after that.* (Researcher, Site C)

PIs spoke about how they raised awareness and promoted the trial before their site opened, and some used team meetings to highlight ongoing challenges with recruitment and encourage anaesthetists to support MAGIC:

*[S6] has gone to their governance meetings and raised that this is going to be happening. And we sent an email to paediatric department as well so the awareness in the trust, we have created quite a bit in the theatres to the surgical unit, recovery.* (PI, Site D)

### Setting- and context-related factors

#### Barriers

There are practical difficulties recruiting on surgical day units/wards, which are described as ‘fast-paced’, ‘high-pressured’ and ‘busy’. This was perceived as a challenge before sites opened:

*How we're going to fit this all in because there's a lot to get done prior to the patient going to theatre and they have very, very strict, you know, time brackets for these patients. […] So it’s always going to be quite difficult to bring people together in a fast-paced environment.* (Research Nurse, Site F)

Anaesthetists were also under pressure from managers not to delay lists and needed to consider the needs of patients more generally, placing limits on recruitment:

*We couldn’t actually have days when it was really, really busy because some days we have more patients than the beds basically so that was one thing that we had to consider that we couldn’t sort of be blocking beds in the study and then we have no beds for the patients.* (RN, Site D)

Individual sites also had specific challenges, relating to the way in which screening and recruitment were organised. It was recognised that ideally participants and parents have a reasonable amount of time to consider taking part in research. If there is no pre-assessment clinic, potential participants are identified when they display anxiety on the day. Given the paperwork involved, research nurses had limited time to decide whether to approach a family:

*You’re initially having to go in and judge a family within five or ten minutes as to whether they’re suitable for the trial or not.* (Research Nurse, Site E)

As noted above (participant-related factors), being anxious can be a barrier, and thus not having the ability to approach patients in advance was seen as a challenge at some sites:

*You’re dealing with a certain cohort of people who are very nervous anyway by the nature of being eligible. I think that just approaching them on the day is often, you know, just too much for them really.* (Researcher, Site C)

Furthermore, patients who were first on the list were particularly difficult to recruit because usual processes might have started before the researchers had a chance to speak to the anaesthetist and approach the patient. Some stakeholders felt it was difficult in these circumstances to change the order of patients on the surgical list

Identifying anxious patients in advance was not always a guarantee they would be eligible for MAGIC. Where sites identified anxious children through pre-assessment clinics and referrals from dentists, this did not always translate into the child needing a pre-medication:

*The ones that are identified by the dentist who come in, it's maybe only 75 percent of those who actually end up getting a pre-med because there's a slight difference between how cooperative a child may or may not be within a dental suite and coming into a children's sort of theatre ward environment.* (Anaesthetist, Site E)

Patients who did require a pre-medication on the day of surgery and did not always appear anxious at pre-assessment.

Obtaining pre-medications from hospital pharmacies can affect recruitment. Several stakeholders reported that walking to the pharmacy took a considerable amount of time, and research pharmacists were not always available for the start of the theatre list. The spaces used for normal practice could also act as a constraint or at least something that needed addressing in order to recruit patients:

*The randomisation requires Wi-Fi. And that, so we've had to wander around the building and identify places where you could potentially get a signal just so randomise somebody. And that might be moving out of the department.* (Researcher, Site C)

Some of these issues may not have been considered when the protocol was being written. Before recruitment starts, a site may also underestimate how much a factor such as a pharmacy opening times will act as a barrier.

#### Enablers

Sites benefitted from the effective organisation of screening and recruitment. Where sites had pre-assessment clinics, or anxious patients were flagged up by referring clinicians, this was seen as helpful:

*Our nursing staff are very much on board. They’ve been given details of how to recruit. Any patient they identify in pre-assessment, they e-mail myself and the research nurses, and we make sure someone’s on site that to pick that child up.* (PI, Site B)

*That’s worked quite well for us because when the dentists say in advance, “I think this person, this person, this person will need pre-medding,” then we know they’re coming so our research nurses are standing on the ward waiting for them.* (PI, Site E)

When approaching on the day, stakeholders also spoke about putting processes in place within their hospitals to ensure potentially eligible patients were not missed:

*We look at the list one week ahead, every week ahead, we look at the list and see if there are any children.* (PI, Site D)

As noted above, staff availability was a key issue, so looking ahead meant diaries could be coordinated.

The ability and willingness of clinicians to be flexible with surgical lists was a key issue that related to collaboration and settings. Where anaesthetists were willing to move patients on the list, this facilitated recruitment:

*They’re good, they’re flexible, they'll move the patients to allow us time to do all our paperwork.* (Research Nurse, Site E)

Processes also involved organisation of paperwork and working with pharmacies to ensure pre-medications were available as quickly as possible:

*We’ve got quite a good process going though because what we’ve been doing is as we’re randomising the patient, we get somebody to ring the pharmacy so that they know we’ve got somebody potentially coming down so they’re watching and waiting, and then they know that they’ve got somebody free to get on with the prescription.* (Research Nurse, Site B)

### Health system-related factors

#### Barriers

Within the National Health Service (NHS), there are high workloads and limited time provided for research:

*The working load and the time constraints are the biggest challenge really in the NHS in general.* (PI, Site A)

As well as individual research nurse availability (see practitioner barriers), the requirements the health system places on research nurses meant that individuals had other responsibilities, which conflicted with the needs of MAGIC:

*We've got to make sure everyone is as efficient as possible. You can't have the luxury of a nurse, of two nurses hovering around pre-screening, floating around for a bit when we need to be also generating income.* (Research Nurse, Site A)

Sites may be affected by staffing decisions and the availability of resources within particular NHS Trusts, rather than just in relation to staff named on the delegation log. The screening log demonstrated that some patients were excluded due to language barriers (see participant-related factors), and it was noted that it could be difficult to provide an interpreter:

*It’s not easy these days. Interpreters, we have telephones, no face-to-face so it’s difficult. At the moment, luckily we’ve not recruited any child with language issues. And we can’t as well.* (PI, Site D)

#### Enablers

There was not much discussion about how the NHS enables recruitment to RCTs such as MAGIC, although participants acknowledge a wider culture that encourages clinicians to participate in research, the existence of clinical research networks that provide research nurses, and the idea that studies can be seen as ‘good for the department’ (PI, Site D).

## Discussion

Recruitment challenges in RCTs are complex, relating to factors such as the intervention and the study design; patients who are potential participants; practitioners responsible for recruiting to the study; logistical arrangements within individual settings; and the wider healthcare systems in which research takes place. This qualitative study has highlighted barriers and enablers to recruitment to a pragmatic trial of anxious children in paediatric anaesthesia. As with other studies, low recruitment reflected a lower than expected number of potential participants, strict eligibility criteria, gatekeeping by practitioners and their colleagues, limited resources and logistical challenges. MAGIC has addressed some of these barriers by broadening eligibility criteria (to include all specialties and younger children), removing the requirement for assent (although it is still encouraged), allowing sites without pre-operative clinics to send information in advance and working with sites to address other issues with recruitment. For instance, sites were reminded that patients with autism or learning difficulties who appeared highly anxious should still be approached, and they were also encouraged to add more anaesthetists to the delegation log.

Some barriers to recruitment identified here are common across RCTs. Overestimating the number of potential participants is a recognised issue in RCTs, known as Lasagna’s law [38, 39]. Although audits reveal the number of pre-medications provided, the process of recruiting for MAGIC takes place in a time-sensitive situation between a patient arriving in hospital and being taken to theatre and relies on the availability of research nurses and anaesthetists and for site pharmacies to be open to provide the pre-medications. Similarly, eligibility criteria is a recognised barrier to RCT recruitment [15], and in MAGIC, stakeholders suggested that other groups of anxious patients requiring pre-medications who could be included. Interviews with stakeholders involved in the trial design demonstrated that the reasons for particular exclusions reflected issues with obtaining assent from very young children, a desire to avoid other confounding factors, and the age range for whom particular measurement scales were validated. Broader eligibility criteria may improve recruitment, but raise ethical and methodological issues that need to be considered. Finally, time constraints on key members of staff acted as a barrier, as in other studies [15].

Reviewing the screening data illustrated these issues, with relatively low numbers of screened patients at some sites, while others had high proportions of non-eligible screened patients and instances were children were not approached due to a lack of research nurse time. In addition, issues highlighted in the results above were listed as ‘other reasons’, included clinician prescribing other pre-medications, pharmacies not being open, the need for an interpreter, a lack of time to recruit and children requiring higher doses of pre-medication. Nevertheless, the ‘other reasons’ collated from the screening logs at different sites indicate inconsistencies in how these were completed. For example, as noted in practitioner-related factors, the PI of site E spoke about missing patients due to a lack of availability. A review of the screening data found that there was only one recorded incident of the PI and sub-PI not being available at this site, so it may be that patients were not screened at other times. However, other sites recorded multiple occasions when no prescriber was available. Clarifying how sites use the screening logs is important for understanding challenges to recruitment and could be explored through further research at multiple sites [40].

A particular challenge for MAGIC was the requirement to recruit anxious children. While the pragmatic nature of the trial was valued, this study population posed various barriers. Firstly, stakeholders suggested that patients and parents may avoid adding extra stress. Although MAGIC was seen as a relatively straightforward trial from a clinical perspective, an RCT adds uncertainty for the anxious patient and their parent. Engaging with researchers and clinicians about a trial might run counter to a child/family’s coping strategy for the day. In relation to this, health professionals were also concerned about the ethics of adding to a family’s stress, and theatre nurses could ask researchers not to approach a family, or anaesthetists could refuse to allow an approach to their patients. Obtaining assent from an anxious child was also sometimes found to be challenging, particularly if children were unwilling to engage with researchers.

This ‘gatekeeping’ relates to the hidden challenge in much clinical research of the conflict between health professionals’ clinical and caring roles, and their responsibilities to recruit for a research project [6, 11, 17]. Stakeholders in MAGIC reported not recruiting or not approaching anxious children due to previous clinical experience or concern as to how well the patient and their family would cope. Stakeholders were also dependent on the collaboration of other anaesthetists and nurses to achieve recruitment. Some of these collaborators were reported to be making decisions to engage in their usual practices, which they felt would work well for their patients (for example, using a particular combination of pre-medications).

Health professionals aim to act in the interest of patients, and this can include adopting a paternalistic approach based on the perception of patient needs [7]. In MAGIC, the trial population was anxious children, and clinicians and researchers needed to approach their parents at a difficult time. Although the desire not to add to a family’s stress is understandable, paternalism can lead to parents being denied the opportunity to make an informed decision about participation for themselves [18]. The process of recruitment requires various tasks, including connecting and engaging gatekeepers in the RCT in order to establish a shared goal [17]. In MAGIC, stakeholders reported how they were able to build on existing relationships, publicise the research and build rapport to ensure that key health professionals were engaged in the trial, and actively helped to find patients or adapted their usual approach to the benefit of MAGIC. It is important that those running and recruiting for trials recognise the need for this engagement work and that this is accounted for at the planning stage.

As a pragmatic trial, MAGIC took place in the context of day-case surgery. Participants reported external pressures to not waste theatre time, due to the expense and delays for staff and other patients. The specific context of day-case surgery within hospitals, and the wider NHS, could impose pressure not to introduce too much uncertainty. The value of predictability, particularly in a time-pressured context, conflicts with the need to carry out research. MAGIC was generally seen as an important study, but where clinical gatekeepers had successful practices using other pre-medications, or non-pharmacological techniques, stakeholders reported an unwillingness to engage with MAGIC. Using an unfamiliar approach, particularly when there are high stakes if it is not successful, can also be a challenge for stakeholders [13]. Again, this points to the potential benefits of engagement work by researchers involved in an RCT, as well as qualitative research as part of feasibility studies to understand opinions and concerns about an intervention [9].

The importance of having experience in a specific setting has previously been identified in relation to recruitment in emergency care [18] and lack of experience on surgical day units is a potential barrier. However, stakeholders in MAGIC were able to draw on wider experience and skills to adapt to working in a different way. Preparation activities were used to facilitate recruitment. Stakeholders reported putting processes in place to maximise staff availability, reduce the time needed to collect the trial drugs from the pharmacy and ensure potential participants were not missed. While study-, population- and context-related factors can be a significant part of recruitment challenges, finding site-specific ways to address potential barriers can be beneficial. Nevertheless, stakeholders acknowledged difficulties in thinking of ways to improve recruitment, resulting in frustration and to some extent reduced motivation.

Organising barriers and enablers in relation to different factors highlights the range of challenges faced by stakeholders recruiting within a multi-site trial, and the different domains that can be targeted to improve recruitment. However, it is also important to recognise that study-, population- and context-specific challenges interrelate across these domains, with barriers reinforcing each other and potentially multiplying their effects (establishing the *extent* to which particular barriers limited recruitment would need further research and analysis). As identified above, a particular challenge for MAGIC was the population of anxious children about to undergo surgery. This acted as a barrier in terms of participant willingness to engage, gatekeeping by nurses and clinicians to avoid making things worse, and pressure within settings and the wider health system to avoid additional uncertainty, and risk delay and non-cooperation. Existing processes had also been designed to make things easier for patients and their families (such as scheduling anxious patients at the start of surgical lists), and stakeholders reported different experiences of adapting these processes to suit MAGIC. Identifying these cross-cutting themes, and the different ways in which they can act as a barrier to recruitment, is important for developing a plan to improve recruitment to an individual RCT.

### Timing of integrated qualitative research within trials

External pilots and feasibility studies allow for a trial protocol and procedures to be operationalised, tested and reviewed before recruitment starts. However, these approaches can add to the costs and time required for a trial to run [9]. Internal pilots are a pragmatic way to address recruitment challenges, although barriers that are identified at this stage can affect recruitment to the extent that it is not feasible for the trial to continue. Inclusion/exclusion criteria and trial processes can be assessed on paper, but in practice, unforeseen issues may arise once a protocol is operationalised. Barriers to recruitment can be identified through qualitative research in an internal pilot. However, central teams and sites also need time to process findings from qualitative research and implement changes.

One potential approach is rapid, or focused, ethnography, which involves using semi-structured interviews, participant observation and document analysis during a brief period to develop a reasonable understanding of a particular context at a moment in time. This approach is valuable as a way of capturing complexity in a short time frame [41]. Rapid ethnography can connect micro-level observations to macro-level contexts [42], such as the impact of institutional working arrangements and national policies on how trials are operationalised. Although there are concerns that rapid ethnography does not allow researchers to fully capture views within a setting, clearly focused research questions, data collection and analysis can generate valuable data [43].

If used prior to sites opening, this approach could be directed to understanding usual practice at sites, the characteristics of patients, actions being taken to prepare for recruiting and anticipated challenges. Alternatively, rapid ethnography could have been used once sites had opened in order to understand how the protocol had been operationalised at different sites. In addition to interviews, focused observations of recruitment settings and document analysis may have helped illustrate site-specific challenges raised in interviews, for example in relation to the layout of particular hospitals or approach to screening and assessing eligibility. Utilising multiple methods and the principles of ethnography (collecting data about what people do in their everyday lives, not just what they say they do) in this study would have likely allowed for a more detailed understanding of how barriers to recruitment were experienced [44]. An ethnographic approach would also allow for a wider range of health professionals to be approached for interviews if deemed relevant by the researcher; for example, the roles of theatre nurses and anaesthetists outside of the trial were highlighted in the stakeholder interviews and would have been useful to interview directly.

### Strengths and limitations

By interviewing both PIs and research nurses across a range of sites involved in MAGIC, as well as stakeholders responsible for trial design, this paper has explored the perspectives of various health professionals who contributed to recruitment. Drawing on an existing framework for barriers and enablers helps to situate the findings of this study in relation to other work on recruitment and potential future qualitative research on barriers and enablers to RCT recruitment.

The topic guide was focussed on identifying issues with recruitment and the delivery of the study that could be addressed in the main trial. The framework used for a more detailed analysis of barriers and enablers to recruitment was identified after the interviews were conducted, and additional questions could usefully have been added on dual clinician/researcher roles and experiences of conducting research within the health system of the NHS. The interviews conducted in this study were detailed discussions that highlighted the complexities of recruiting to a pragmatic paediatric trial within anaesthesia.

As the analytical framework was applied after data collection, it is not possible to say that saturation was achieved. Nevertheless, a range of barriers and enablers were identified across the domains. The analysis also indicated that certain barriers were particularly significant to stakeholders, and this has been highlighted in the discussion section of this paper.

Interviews were conducted with stakeholders involved in MAGIC. Additional research with other clinicians, health professionals and site managers would have been useful, in order to gain an insight into wider views on MAGIC and the way the study impacted on day-to-day practices.

## Conclusion

There are opportunities to increase recruitment to RCTs if potential study-wide and site-specific challenges can be identified and addressed early in the process. Qualitative research with stakeholders to identify concerns with the protocol and the extent of preparation activities could help to identify potential barriers to recruitment before sites open.

Study-, population- and context-related factors reflect potential difficulties inherent within a particular protocol, that are realised through its operationalisation by practitioners and other health professionals with whom they collaborate, in specific clinical settings. There are multiple barriers to recruiting anxious children due to undergo surgery, including parents, practitioners and other health professionals not wanting to make anxiety worse, or make things more difficult for families. Recruiting children due to undergo surgery from a surgical day unit is also challenging, due to the busy environment, and pressures internalised by clinicians and imposed within settings and the wider health system.

Where the aim of a trial is viewed as valuable, recruitment can be enabled by various aspects of engagement work and effective processes between clinicians, researchers and health professionals whose day-to-day work is affected by the study. Successful communication, preparation and ongoing work to address issues are all useful.

Recruitment within multi-site trials reflects different combinations of barriers and enablers in relation to different domains, and understanding how these interrelate and reinforce one another is important for developing strategies to improve recruitment.

### Recommendations

Trial teams should account for the need for research nurses to carry out engagement work and other preparation activities before sites open. Depending on the trial, this may involve conversations with other health professionals, observations of usual practices in recruitment settings and developing standard operating procedures for all recruiters.

Trial teams and Principal Investigators should consider the prior experience of recruiting staff, with regard to setting and population. Trials could include provision for clearly defined mentoring relationships between researchers, including across sites.

Trial teams should consider building in additional qualitative research during trial set-up. Rapid ethnography could be used to gain an understanding of how sites usually work in practice, the characteristics of their patients, extent of engagement work and anticipated challenges to recruitment.

Trial teams should consider using ethnographic research to understand how sites are operationalising the protocol, including interviews, observations and document analysis. Trial teams should also consider building in time to reflect on findings from qualitative research conducted during an internal pilot and to implement changes.

## Supplementary Information

**Additional file 1.** MAGIC Pilot Study, Topic Guide for Stakeholder Interviews.

## Data Availability

The datasets analysed during the current study are available from the corresponding author on reasonable request.

## Abbreviations

CI: Chief investigator
ENT: Ear, Nose and Throat
GCP: Good Clinical Practice
IMP: Investigational Medicinal Product
MAGIC: Melatonin for Anxiety prior to General anaesthesia In Children
mYPAS: Modified Yale Preoperative Anxiety Scale
NHS: National Health Service
PI: Principal Investigator
RCT: Randomised control trial
