# Supplementary material for: Stakeholder perspectives on barriers and enablers to recruiting anxious children undergoing day surgery under general anaesthetic: a qualitative internal pilot study of the MAGIC randomised controlled trial
Source: Trials. 2021 Jul 16;22:458. doi: 10.1186/s13063-021-05425-z (PMC8285773; doi:10.1186/s13063-021-05425-z)
Supplement: Supplementary file 1 — Additional file 1. MAGIC Pilot Study, Topic Guide for Stakeholder Interviews. [file 13063_2021_5425_MOESM1_ESM.docx]

MAGIC Pilot Study: Topic Guide for Stakeholder Interviews

Warm-up questions

Could you start by introducing yourself and telling me about your involvement in MAGIC [establish responsibilities of participant and experience of MAGIC pilot trial].

**Pilot trial [adapt relating to specific job role of participant]**

I want to understand how well MAGIC is working in different hospitals and how this pilot trial could have been improved. What’s your impression of the pilot study at this stage?

- Is there anything you think is working particularly well?
- Have you encountered any problems?

[Follow up questions depending on answers]

What are your experiences of recruiting participants into MAGIC?

Have you faced any challenges recruiting participants into MAGIC?

- Prompt for demographic differences among children/parents

Have parents or children raised any questions with you about MAGIC?

- Prompt for understanding of randomization, any challenges explaining randomisation

What do you think of the information sheets being provided to parents?

What do you think of the information sheets being provided to young children?

What do you think of the information sheets being provided to older children?

What do you think of the video for children?

Is there anything about the information that you think needs to be changed?

What communication have you had with the trial management team?

Is there anything you think could be improved about the communication?

Have any issues emerged that you needed to contact the trial management team about?

Are there any other ways the trial management team could support you during the trial?

Could you tell me about how MAGIC is organized in this hospital, how does it work on a day-to-day level?

Is there anything you’ve done here that’s worked particularly well?

Have you had any difficulties following the protocol?

Have you had any challenges administering the premedications?

Have you had any difficulties following up with participants after they leave hospital, how have parents responded to the two week follow up call?

Is there anything else you want to tell me about your experience of the pilot trial?

**Evaluation of their training about MAGIC**

Could you tell me about the training you received before taking part in MAGIC?

- Where did the training take place (i.e. was it face-to-face or online)?
- What was covered in the training?
- Was there anything that wasn’t clear?
- What did you think about how the training was delivered?

How prepared did you feel for the pilot trial following the training?

What did you find helpful about the training?

What could be improved about the training?

**Main trial [questions relating to the main trial will depend on participant’s role]**

The MAGIC team would like to learn from the pilot trial in order to improve the main trial.

From your experience of the pilot trial, do you think any changes need to be made to the main trial? [refer back to any issues raised earlier in the interview]

- Is there anything that needs to be considered about how sites are recruited?
- Is there anything that would encourage sites to take part in the main trial?
- Is there anything about the protocol that you think would discourage a hospital from taking part in the main trial?
- Are there any changes you would make to the training before the main trial?
- Is there anything else the research team could do that would help hospitals to prepare for taking part in the trial?
- Are there any changes that could be made to the protocol that would help you to recruit patients?
- Is there anything that could be improved about the information for parents?
- Is there anything that could be improved about the information for young children?
- Is there anything that could be improved about the information for older children?
- Is there anything else the research team could do to support hospitals involved in the main trial?
- Is there anything you’ve learnt from the pilot trial that you think it would be useful for other hospitals to know about?

**Comparison with other research**

The team would like to understand how MAGIC compares to other research studies? What other research have you been involved in prior to MAGIC?

How does MAGIC compare to other research you’ve been involved in?

How did you find recruiting to MAGIC compared to other trials?

How did children respond to MAGIC compared to other trials?

How did parents respond to MAGIC compared to other trials?

Have you learnt anything from previous trials that you think would be useful for MAGIC?

#### Closing

Participants will be asked if they would like to add any further information and thanked for the discussion.
